# Effect of Cold Swaging on the Bulk Gradient Structure Formation and Mechanical Properties of a 316-Type Austenitic Stainless Steel

**DOI:** 10.3390/ma15072468

**Published:** 2022-03-27

**Authors:** Dmitrii Panov, Ruslan Chernichenko, Egor Kudryavtsev, Denis Klimenko, Stanislav Naumov, Alexei Pertcev

**Affiliations:** 1 Laboratory of Bulk Nanostructured Materials, Research Institute of Materials Science and Advanced Technologies, Belgorod State University, 85 Pobeda Str., 308015 Belgorod, Russia; chernichenko@bsu.edu.ru (R.C.); kudryavtsev@bsu.edu.ru (E.K.); klimenko@bsu.edu.ru (D.K.); naumovstanislav@yandex.ru (S.N.); 2 Department Chief Metallurgist, Perm Scientific-Research Technological Institute, 41 Geroev Khasana Str., 614990 Perm, Russia; perets_87@mail.ru

**Keywords:** gradient structure, austenitic stainless steel, swaging, finite element simulation, texture, strength, ductility

## Abstract

The present study aimed to discover the effect of cold swaging reduction on the bulk gradient structure formation and mechanical properties of a 316-type austenitic stainless steel. The initial rod was subjected to radial swaging until 20–95% reduction of initial rod diameter, at room temperature. According to finite element simulation, higher plastic strain was accumulated in the surface layer compared to the center region during swaging. Microstructural investigations revealed three-stage gradient structure formation in the center and edge regions of the deformed rod. Meanwhile, cold swaging resulted in the development of strong 111ǁBA, 001ǁBA, and weak 111ǁBA texture components in the center and edge, respectively. Significant tensile strengthening was observed after cold swaging. For instance, the yield strength (YS) increased from 820 MPa to 930 MPa after 40–80% reduction respectively, without the loss of ductility (δ–14%). This unique aspect of the mechanical behavior was attributed to the gradient structure of the cold swaged material and explained in detail.

## 1. Introduction

Austenitic stainless steels demonstrate an excellent combination of ductility, corrosion resistance, and workability [1,2]. However, low yield strength restricts some of their structural applications. To improve mechanical properties, the steels are strain-hardened which usually reduces their ductility [3,4,5] and impact toughness [6]. Therefore, the obvious strength–ductility trade-off is a challenging issue to achieve high mechanical properties.

Simultaneous development of the yield strength and ductility is possible using some recent strategies [7,8,9]. For instance, producing a heterogeneous lamellar austenitic structure in the 316L steel, consisting of lamellar recrystallized grain clusters and lamellar coarse grains, results in a satisfactory strength–ductility combination [8]. Analogically, the design of a heterogeneous austenitic structure with micro- and nano/ultrafine grains is also associated with a good synergy of high strength and ductility [10]. On the other hand, the 316L steel with an excellent combination of ultimate strength (1000 MPa) and ultimate elongation (27%) is prepared via the development of a heterogeneous structure containing ductile recrystallized grains, hard nano-sized twins, and nano-grains [11]. Various grain size relates to different austenite stability that improves strain-hardening ability through multi-stage transformation induced plasticity [12,13].

Meanwhile, producing a gradient structure, defined as the gradual change of the phase composition, grain size, or phase morphology along the cross-section or length of the workpiece seems to be another promising pathway of enhancing mechanical properties. Therefore, copper with the gradient structure demonstrates ductility like copper with a coarse grain structure and sufficiently enhanced strength characteristics [7]. In austenitic stainless steels, the gradient structure has been often realized by torsional [14] or surface deformation [12] in thin surface layers (~200 μm). In surface layers of the AISI 304 steel, austenitic domains with various dimensions from the surface to the center have been derived by the friction treatment that also performs an excellent combination of strength and ductility [14]. For the AISI 316L steel, the gradient structure obtained by surface mechanical rolling treatment results in the significant improvement of both low and high fatigue properties due to the inhibition of crack nucleation and accommodation of a remarkable cyclic plastic strain amplitude [15]. On the other hand, surface mechanical rolling treatment and following annealing of the AISI 316L steel may enhance strength–ductility synergy and corrosion resistance simultaneously [16].

It is worth noting that the main body of studies is devoted to materials with a gradient structure characterized by a few hundreds of micrometers in depth. Therefore, the bulk gradient structure in austenitic stainless steels is not studied systematically regarding the influence of their structure on mechanical properties. Recently, it has been presented that the bulk gradient structure in austenitic stainless steels might be obtained by cold radial swaging [17] because of the non-uniform plastic strain accumulation in the cross-section [18]. The present study aimed to discover the effect of cold swaging on the gradient structure formation and mechanical properties of the 316-type austenitic stainless steel.

## 2. Materials and Methods

### 2.1. Material Processing

The program material was the commercial 316-type austenitic stainless steel with the following chemical composition (wt.%): Fe–0.08%C–16.4%Cr–12.3%Ni–2.18%Mo–1.28%Mn–0.42%Si. To obtain an as-received rod with 46 mm in diameter, the initial ingot was subjected to hot radial swaging at 1200 °C and subsequent annealing at 1100 °C for 2 h with water cooling. Then, the as-received rod underwent cold radial swaging at room temperature using an SXP-16 radial forging machine with four radially moving hammers with the following mode: workpiece feeding rate 180 mm/min, stroke frequency 1000 blows per minute, workpiece rotation speed 25 rotations per minute. The water cooling of the rod was realized during swaging. Swaging was performed in five steps of reduction: 20%, 40%, 60%, 80%, and 95%.

### 2.2. Finite Element Simulation

Finite element simulation (FES) of swaging was carried out using QFORM software (V. 9.0.7, QuantorForm, Moscow, Russia). The basic model included four hammers that performed the deformation and a pusher that provided the axial and rotational movement of the workpiece during processing. An adaptive finite element net was used. The size of the finite elements decreased with proportionality coefficients in the range from 3 to 15. The maximum and minimum size of the meshes were 6 mm and 0.2 mm, respectively. The total number of the meshes varied from 20,000 to 197,000. The heat transfer between hammers and the workpiece was calculated only on the contact surface. The resulting heat transfer coefficient at the contact surface was 2500 W/m^2^K. Friction between the rod surface and hammer was set by Levanov’s law [19] with a Levanov’s coefficient of 1.25 and a friction factor of 0.8. Water with a constant temperature of 40 °C was used as the environment to reduce the heating of the workpiece. The technological parameters of swaging were similar to the applied swaging modes (Section 2.1).

### 2.3. Microstructural Observations

Transmission electron microscopy (TEM) and scanning electron microscopy (SEM) were applied to characterize the microstructure. TEM was performed on thin foils using a JEOL JEM-2100 electron microscope (JEOL, Akishima, Tokyo, Japan) with an accelerating voltage of 200 kV. Flat templates with a thickness of 0.3 mm were cut in transverse and longitudinal rod cross-sections by a Sodick AQ300L wire electrical discharge machine (Sodick Co, Fukui, Japan). Then, the templates were exposed to double-sided grinding by sandpapers to 0.1 mm thickness. Subsequent conventional twinjet electro-polishing of discs with 3 mm in diameter was carried out using TenuPol-5 (Struers, Ostrava, Czech Republic). The electrolyte consisted of 5% perchloric acid, 35% butanol, and 60% methanol. The microstructural parameters were estimated on bright- and dark-field TEM images.

SEM observations were conducted on the thin foil surface using FEI Nova NanoSEM 450 scanning electron microscope (FEI Company, Hillsboro, OR, USA) equipped with an EDAX Hikari EBSD camera (EDAX, Mahwah, NJ, USA). EBSD maps were received with a step of 200 nm. Backscattered electron diffraction patterns were analyzed using the TSL OIM Analysis software (V. 8.1, EDAX, Mahwah, NJ, USA). To improve the quality of EBSD data, points with the confidence index (CI) ≥ 0.1 were taken for the subsequent analysis.

X-ray diffraction (XRD) analysis was conducted on microsections in a transverse cross-section of the rod using a Rigaku Ultima-IV X-ray diffractometer (Rigaku, Akishima, Tokyo, Japan) in CuKα-radiation. Scanning was carried out in standard Bragg–Brentano geometry in the 2Θ angle range from 40 to 100° with a step of 0.02°.

To estimate the content of the magnetic α-phase in the center and edge of the rod, a FERRITSCOPE FMP30 eddy-current tester (Helmut Fischer Holding GmbH, Sindelfingen-Maichingen, Germany) with the converter detector FGAB1.3-Fe was used. The tester was calibrated by standard samples. Five measurements were made and averaged.

### 2.4. Determination of Mechanical Properties

The Vickers microhardness testing was performed by a Wolpert 402MVD semi-automatic hardness tester (Wolpert, Maastricht, Netherlands) equipped with a diamond pyramid indenter with a base angle of 136°. The measurements were performed along diameter in transverse cross-sections with a load of 10 g and a dwell time of 15 s. Two perpendicular testing paths were realized for each sample with subsequent averaging of the results.

Tensile tests were conducted at room temperature and the strain rate of 1 × 10^−3^ s^−1^ using Instron 5882 (Instron, Norwood, MA, USA), Instron 300LX (Instron, Norwood, MA, USA), and Metrotest RGM-600 (Metrotest, Moscow, Russia), electromechanical testing systems depending on the required maximum load of 100, 300, and 600 kN, respectively. Flat tensile specimens were cut from the central part of the rod along the axial direction with the gage width of $b_{0}=0.83D_{0}$, the gage thickness of $t_{0}=0.19D_{0}$, and the gage length of $l_{0}=5.65\sqrt{S_{0}}$, where $D_{0}$ is the diameter of a rod and $S_{0}$ is the cross-sectional area of a specimen. The elongation during testing was estimated by the digital image correlation (DIC) technique using a VIC-3D non-contact system (Correlated Solutions, SC, USA). Data processing was carried out using the VIC 2D software (V. 60.0.665, Correlated Solutions, SC, USA).

## 3. Results

### 3.1. Effect of Cold Swaging on the Bulk Gradient Structure Formation

#### 3.1.1. As-Received Structure

**Figure 1:**
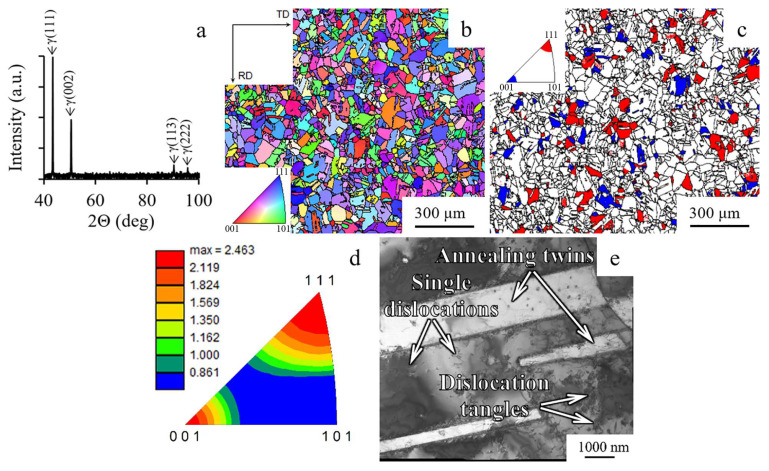
(**a**) XRD pattern, (**b**) inverse pole figure map, (**c**) texture map, (**d**) inverse pole figure, and (**e**) TEM-structure of the as-received program steel.

The XRD profile of as-received material indicated a single-phase face-centered cubic (FCC) structure, as shown in Figure 1a. Microstructural investigation using EBSD-analysis confirmed equiaxed grains of austinite phase having an average size of ~55 μm, as shown in Figure 1b. Meanwhile, the fraction of Σ3-type boundaries reached 46%, which was associated with an annealed state. The weak initial crystallographic texture with the 001ǁrod axis (RA) and 111ǁRA components was found (Figure 1c,d). The fraction of grains with the texture of 001ǁRA and 111ǁRA was 7–10% and 10–14%, respectively (Figure 1c). According to the inverse pole figure (Figure 1d), the 001ǁRA and 111ǁRA texture components reached 2–2.5 MRD (multiple of random distribution). Within austenitic grains, wide annealing twins, single dislocations, and dislocation tangles were found (Figure 1e). However, a cell structure was not observed.

#### 3.1.2. Phase Content Evolution

**Figure 2:**
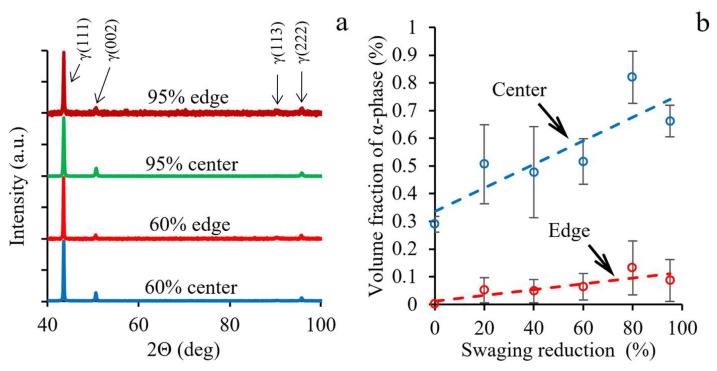
(**a**) X-ray patterns and (**b**) α-phase fraction after different swaging modes. The dashed lines in 2b are only guides to readers.

X-ray patterns and results of eddy-current testing after different swaging reductions are presented in Figure 2. After a 60% and 95% reduction, only γ-phase reflections were found in X-ray patterns of the center and edge (Figure 2a). In comparison to the as-received state (Figure 1a), the intensity of the (002)γ reflection significantly decreased. In the center, the (002)γ reflection was about twice that at the edge. Meanwhile, the relative intensity of the (222)γ reflection remained almost unchanged. These differences were caused by the variation in texture between these parts of the rod that was presented in detail in Section 3.1.3.

Despite the absence of α-phase reflections in X-ray patterns of the cold-swaged rod (Figure 2a), a small amount of ferromagnetic α-phase was detected by the eddy-current method (Figure 2b). The common fraction of α-phase did not exceed 1%, yet, in the center, the fraction was more in comparison to the edge. Interestingly, the average volume fraction of α-phase increased as a function of increasing swaging reduction.

#### 3.1.3. EBSD Analysis

**Figure 3:**
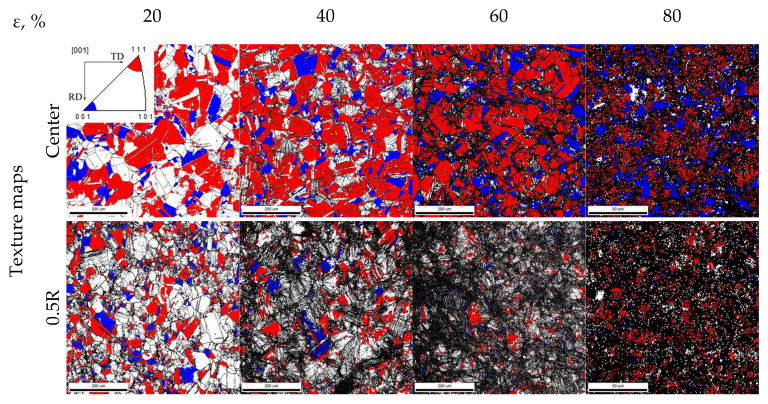

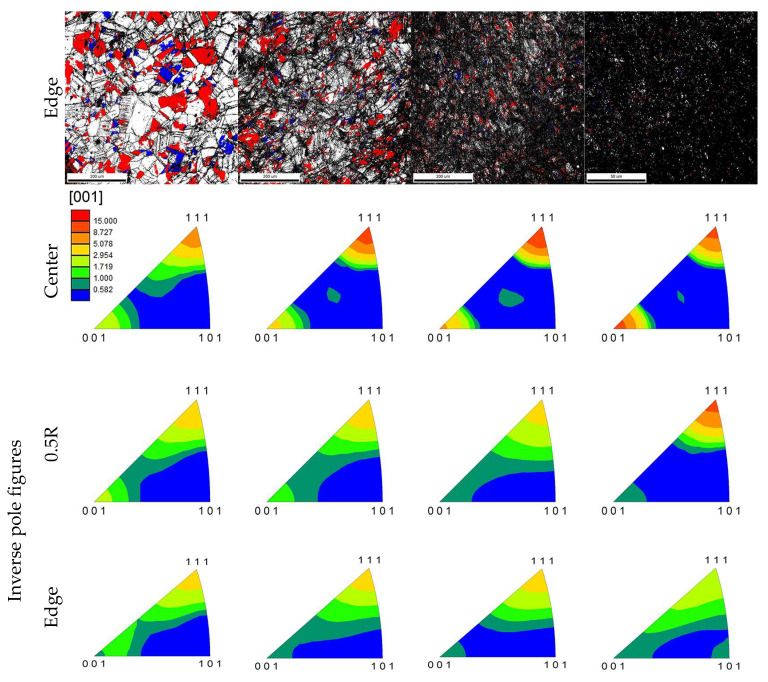
Texture maps and corresponding inverse pole figures after different swaging modes.

Texture maps and inverse pole figures after different swaging modes are presented in Figure 3. According to the EBSD analysis, noticeable changes of the texture in the radial direction were obtained. After a 20% reduction, the texture in the center increased to ~7.3 and ~2.7 MRD for the 111ǁRA and 001ǁRA components, respectively. At the edge, the intensity of the 111ǁRA and 001ǁRA components was 3.8 and 1.2 MRD, respectively. In the center, an increase in a reduction to 60% was associated with the significant sharpening of the 111ǁRA component to 14 MRD. However, an 80% reduction resulted in decreasing this texture component to 11.95 MRD. Meanwhile, the intensity of the 001ǁBA component increased continuously to 10.81 MRD. On the other hand, at the edge, the texture intensity was 2–5 MRD after all applied swaging modes. Furthermore, the weak one-component 111ǁBA texture formed after a 40% reduction.

**Figure 4:**
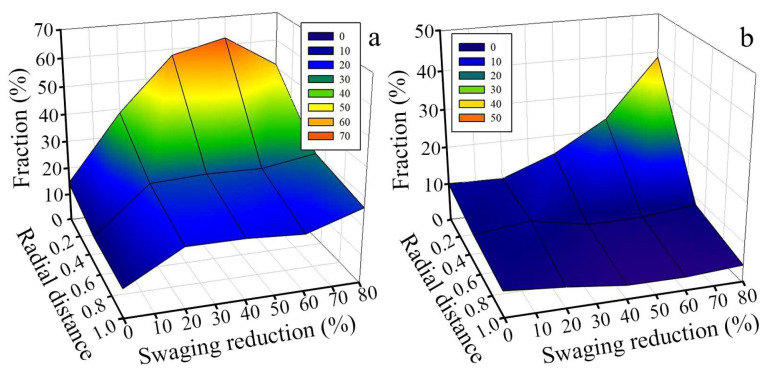
Effect of swaging reduction on the distribution of the volume fraction of grains with (**a**) the 111ǁRA and (**b**) 001ǁRA texture components.

The effect of swaging reduction on the distribution of the volume fraction of grains with the 111ǁRA and 001ǁRA texture components are shown in Figure 4. The following trends were associated with the evolution of the texture: (i) the total fraction of 111ǁRA-oriented grains in the center reached a maximum of 63% after a 60% reduction with a following decrease to 51% after an 80% reduction (Figure 4a), while the fraction of 001ǁRA-oriented grains steadily increased (38.6%) with increasing swaging reduction to 80% (Figure 4b); (ii) the common fraction of 111ǁRA- and 001ǁRA-oriented grains at the edge did not exceed 24–27%.

#### 3.1.4. TEM Observations

**Figure 5:**
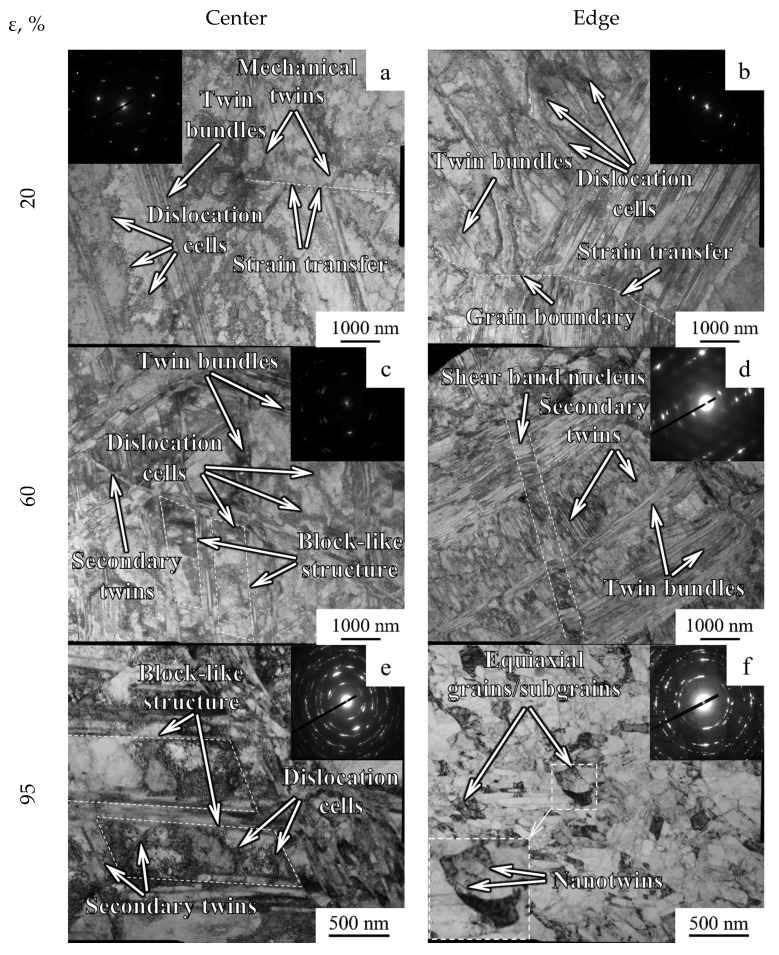
Transverse TEM structure in (**a**,**c**,**e**) the center and (**b**,**d**,**f**) edge after different swaging modes: (**a**,**b**) 20%, (**c**,**d**) 60%, (**e**,**f**) 95%.

Transverse TEM structures are presented in Figure 5. After a 20% reduction, irregular-shaped dislocation cells with several micrometers in width and negligible misorientation were found (Figure 5a,b). Furthermore, mechanical twins were also found in the structure that, according to microdiffraction analysis and EBSD-analysis, were in the {111} plane and exhibited a misorientation of ~60° with a matrix. Apparently, twinning developed over several systems. Typically, mechanical twins were assembled in bundles. It is worth noting that, in some cases, the strain was transferred through the boundaries by inducing twinning within adjacent regions (Figure 5b). However, wide twin bundles might suppress the twinning–twinning strain transfer. In this case, twinning–twinning strain transition means activation twinning within adjacent regions by a certain twin [20]. In comparison to the center, the dislocation cells at the edge became more pronounced and dispersed, on the one hand. On the other hand, there was a significant increase in the width of the twin bundles (Figure 5b).

Swaging with the reduction of 40–80% was associated with the enhancement of secondary twinning within regions bordered by twin bundles that resulted in the block-like structure formation (Figure 5c). The block-like structure consisted of blocks with parallelepiped shape that was formed by twins of two different twinning systems. Meanwhile, the dislocation cells were reduced to ~100 nm therein. At the edge, the fragmentation of the lamellar structure by transverse dislocation boundaries occurred (Figure 5d). According to TEM observations of the longitudinal section (Supplementary Materials Figure S1a), two types of structural elements were observed mostly in the central part: thin and wide lamellae with a width of ~50 nm and 500–1000 nm, respectively. Within wide lamellae, secondary twinning was developed. Furthermore, at the edge, shear banding with an angle of ~30° to lamellae was observed in longitudinal-section TEM images (Supplementary Materials Figure S1b). The shear bands width reached ~1 μm. Within the shear bands, the refined structure was attained.

After a 95% reduction, the transverse block-like structure of the center was fragmented by dislocation cells (Figure 5e). At the edge, transverse dislocation boundaries were observed within the twin-matrix structure (Figure 5f). However, new equiaxial sub-grains with nanotwins inside were observed therein. In the center of the longitudinal section, both thin and wide single lamellae were oriented along the rod axis (Supplementary Materials Figure S1c). At the edge, the thickness of thin and wide lamellae was ~50 nm and ~300 nm, respectively (Supplementary Materials Figure S1d).

### 3.2. Effect of Cold Swaging on Mechanical Properties

#### 3.2.1. Microhardness

**Figure 6:**
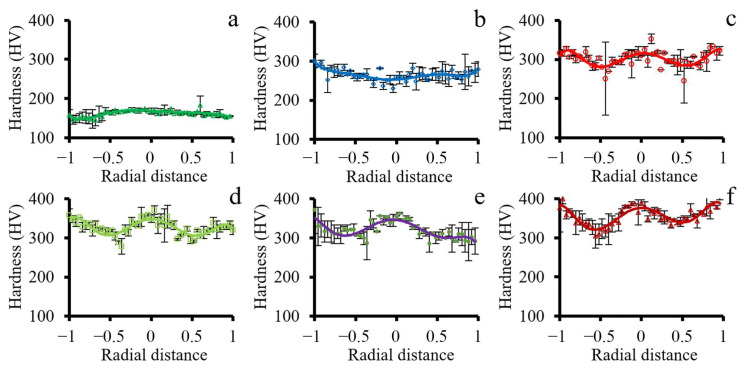
Vickers hardness distribution in the transverse cross-section in (**a**) the as-received condition and after a swaging reduction of (**b**) 20%, (**c**) 40%, (**d**) 60%, (**e**) 80%, and (**f**) 95%.

The microhardness distribution in the transverse cross-section after different swaging modes are presented in Figure 6. In the as-received condition, hardness was uniformly distributed at the level of 150–160 HV (Figure 6a). After a 20% reduction, the hardness of the central part increased to ~250 HV (Figure 6b). Meanwhile, the slight rising of hardness to 280–300 HV was also detected in the direction from the center to the edge. The further swaging reduction caused an increase in the overall hardness level (Figure 6c–f). However, the maximum in the central part of the cross-section was obtained. In the radial direction, the minimum was derived at half the radius. Yet, the hardness level tends to increase towards the edge. Swaging with a 95% reduction was associated with the highest hardness level of ~380 HV in the center and edge (Figure 6f).

#### 3.2.2. Tensile Testing

**Figure 7:**
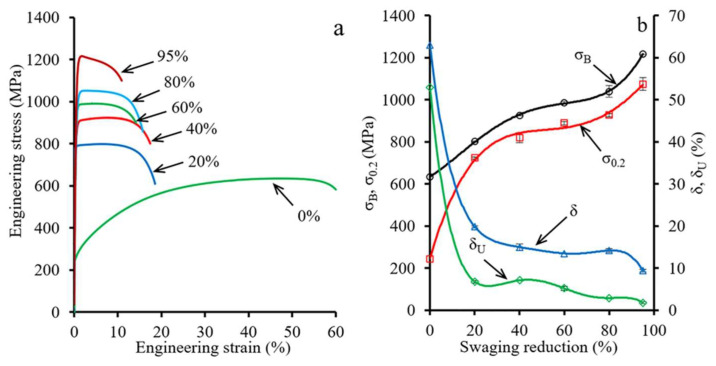
(**a**) Stress-strain curves and (**b**) tensile properties-strain plots of the program steel. Notation: σ_B_—Ultimate tensile strength; σ_0.2_—Yield strength; δ—Elongation to failure; δ_U_—Uniform elongation.

Engineering stress-strain curves and tensile properties-swaging reduction plots are shown in Figure 7. In the case of the as-received condition, a typical tension diagram with a large uniform elongation (δ_U_~45%) was observed (Figure 7a). After a reduction of 20%, uniform elongation (δ_U_) decreased dramatically to 7%. However, after a 40–80% reduction, the uniform elongation substantially decreased from ~7 to ~3%. Meanwhile, after a 40% reduction, the elongation to failure (δ) dropped from 67% to ~14% with the following saturation up to an 80% reduction. Further reduction of 95% caused a slight decrease in the elongation to failure to ~9%. Furthermore, an increase in the ultimate tensile strength (σ_B_) up to 1220 MPa after a reduction of 95% was detected (Figure 7b). Yield strength (σ_0.2_) exhibited similar behavior.

## 4. Discussion

### 4.1. Finite Element Simulation of Swaging Processing

The presented results of the structure characterization demonstrated various texture patterns, structure condition, and difference in the phase content between the center and edge after different swaging reductions (Section 3.1). Such differences might be associated with non-uniform stress distribution and plastic strain accumulation of various rod layers during swaging.

**Figure 8:**
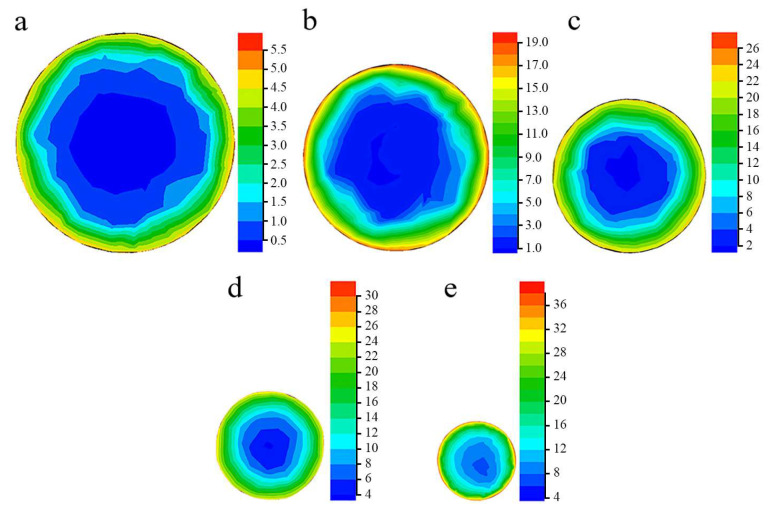
Effect of swaging with a strain of (**a**) 20%, (**b**) 40%, (**c**) 60%, (**d**) 80%, and (**e**) 95% on strain distribution in the transverse cross section.

**Figure 9:**
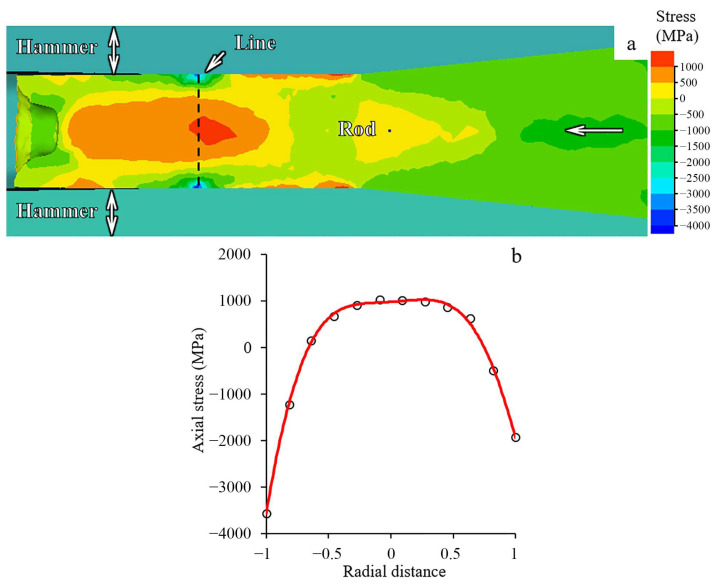
(**a**) Map of axial stress distribution and (**b**) axial stress-radial distance plot during swaging of the rod on the last step (a 95% reduction). Stress distribution in Figure 9b was estimated along the line in Figure 9a.

To clarify the reason for the obtained gradient structure, finite element simulation (FES) was applied (Figure 8 and Figure 9). According to FES, a 20% reduction resulted in the strain accumulation mainly in the near-surface layers (Figure 8a). As the reduction increased, inhomogeneous strain accumulation developed over the transverse cross-section (Figure 8a–d). Thus, the swaging processing was associated with the formation of the pronounced strain gradient in the radial direction that was enhanced as the swaging reduction increased. It is significant to note that the strain distribution was nearly axisymmetric in relation to the rod center. Apparently, high compression axial stresses were derived in the surface layers of the rod (Figure 9). Meanwhile, in the center, an area of moderate tensile and compressive tensile axial stresses was observed.

Interestingly, FES predicted heating the workpiece during swaging (Supplementary Materials Figure S2). During the first (a 20% reduction) and second step (a 40% reduction) of swaging, the surface layers of the workpiece were heated to ~200 and ~300 °C, respectively. The third and following steps were also accompanied by heating to ~350 °C. However, outer water cooling resulted in a slight decreasing temperature of the workpiece surface.

### 4.2. Gradient Structure Formation

According to FES (Section 4.1), increasing swaging reduction resulted in the accumulation of non-uniform plastic strain throughout the rod cross-section. Thus, the higher plastic strain was attained in the surface layers. Furthermore, moderate tensile and compressive axial stresses were obtained in the center during swaging (Figure 9a). Meanwhile, at the edge, high axial compressive stresses were derived (Figure 9b). On the other hand, heating of the rod up to 350 °C was also predicted by FES (Supplementary Materials Figure S2). Hence, such features of swaging resulted in the gradient structure formation. To understand the mechanisms of the structural gradient formation, the structure and texture evolution should be analyzed in detail.

As was established earlier [21,22,23], deformation mechanisms depended on the stacking fault energy (SFE). Low SFE (<13 mJ/m^2^) promoted the formation of strain-induced ε-martensite, whereas in the range of 13 < SFE < 18 mJ/m^2^, the formation of α-martensite was favored over ε-martensite. Twinning was expected when SFE was in the interval of 18–45 mJ/m^2^. In the case of SFE > 45 mJ/m^2^, dislocations glide controlled the plastic deformation.

To estimate the SFE level of the program steel, the following equation for water-cooled from 1050 °C Fe-Cr-Ni austenitic stainless steels was applied [24]:

(1) SFE=−7.1+2.8×Ni (pct)+0.49×Cr (pct)+2.0×Mo (pct)−2.0×Si (pct)+0.75×Mn (pct)−5.7×C (pct)−24×N (pct),

where Ni (pct), Cr (pct), Mo (pct), Si (pct), Mn (pct), C (pct), and N (pct) are weight percentages of corresponding elements (Section 2.1). SFE of the program steel was calculated at 39.39 mJ/m^2^ which satisfied the twinning development.

#### 4.2.1. Gradient Texture Evolution

During swaging, competition between dislocation slide and twinning occurred. Furthermore, it might also be associated with competition between different glide systems [25]. However, according to Ref. [26], at a low strain (below a 20% reduction), the primary deformation mechanism of the program steel might be dislocation sliding. In this case, dislocation sliding possessed the lower critical resolved shear stress in comparison to twinning [27]. On the other hand, twinning was obtained in single-crystal stainless steel 316L with [111] orientation after low strain, while, in the case of [001] and [123] orientations, twinning was only detected after at least a 10% strain [28]. After twinning saturation of [111]-oriented crystals, cell formation occurred between lamellae during subsequent deformation. Therefore, strengthening during swaging with a 20% reduction was associated with dislocation cell development and subsequent twinning (Figure 5a,b). The twinning development in the center resulted in the formation of mostly detached twin bundles.

**Figure 10:**
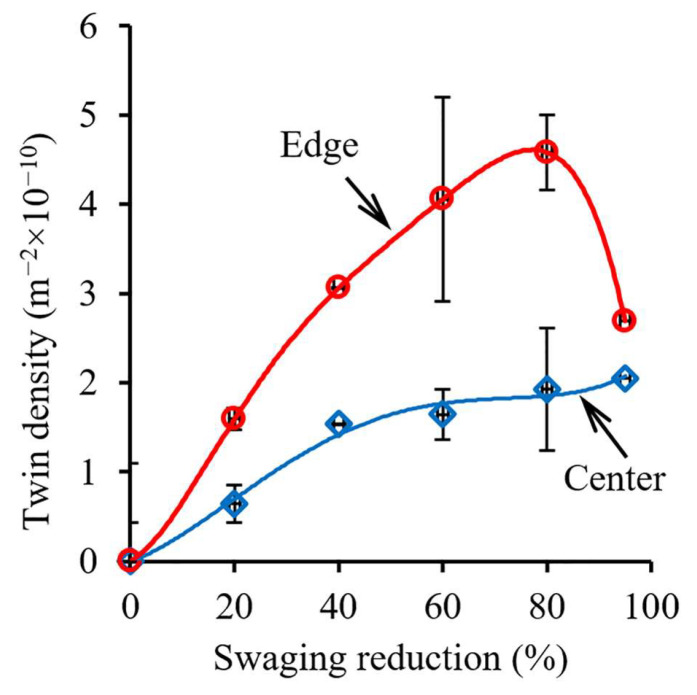
Effect of strain on twin density.

After a 60% reduction, a block-like structure was found in the center due to multiple twinning in the majority of grains (Figure 5c). Meanwhile, the total fraction of 111ǁRA-oriented grains increased to 63% therein (Figure 4a). According to [29], the development of the 111ǁRA texture might be associated with the effect of the dislocation sliding. However, due to further strengthening during 60 → 80% reduction, twinning development in the center (Figure 10) might have resulted in increasing the fraction of 001ǁBA-oriented grains and decreasing the fraction of 111ǁBA-oriented grains (Figure 4) that agreed with the results of Ref. [29]. On the other hand, at the edge, intensive twinning and simultaneous dislocation slide (Figure 5d) were accompanied with the development of the weak one-component 111ǁBA texture after a 40–60% reduction. The fine structure with high density of lattice defects might result in a large number of pixels with a low confidence index (CI < 0.1) that were deleted from considered texture maps (Figure 3). The latter might also decrease the texture intensity.

Meanwhile, the nucleation of shear bands after a 40–60% reduction was also observed (Figure 5d) which was associated with inhomogeneous plastic deformation. In the refined lamellar structure of materials with medium or low SFE, such shear bands appeared because it was difficult to diffuse all applied strain by dislocation sliding and twinning [30]. On the other hand, bidirectional γ → α’ → γ transformation might also result in the shear band formation during cold rolling with vectors of reorientation of θ ≈ 60°〈110〉 and θ ≈ 35°〈110〉 [31,32,33] that might be associated with a negligible amount of retained α-phase (Figure 2b).

Apparently, a trend to saturation of twinning in the edge provoked activation of shear banding therein (Figure 5d). According to Ref. [26], the onset of shear banding of the 316L steel was derived after a rolling reduction of 30–50%. Moreover, as was established earlier [34], shear bands developed due to non-uniform dislocation sliding within the matrix and twins. Thus, multiple shear banding resulted in the structure refinement. In the current study, shear bands intersected the twin-matrix structure with an angle of ~30° to lamellae in longitudinal cross-sections and possessed a width of ~1 μm (Supplementary Materials Figure S1b). Furthermore, shear banding occurred especially at the edge where high compression axial stresses were expected in comparison to the center where moderate tensile axial stresses were attained (Figure 9).

#### 4.2.2. Recovery and Recrystallization Development

As was established elsewhere [35], the recrystallization temperature was 0.4–0.7 of the melting point (for the 316 steel, the melting point is 1375–1400 °C). Hence, the program steel possessed the recrystallization temperature of 386–898 °C. Moreover, the recovery and recrystallization temperatures tended to decrease with an increase in lattice defect density [36]. Therefore, the recovery temperature was below the calculated recrystallization temperature. Thus, recovery and recrystallization additionally might be activated during the late swaging step due to heating to 350 °C (Section 4.1 and Supplementary Materials Figure S2). As was estimated using post-mortem TEM, the twin density increased up to an 80% reduction (Figure 10). After a 95% reduction, the structure of the edge was mostly fragmented by transverse dislocation boundaries (Figure 5f) that also resulted in decreasing twin density. Meanwhile, uniaxial grains/sub-grains were also observed therein. Apparently, such features were associated with the development of dynamic recovery and recrystallization.

Interestingly, within the defect-free grains/sub-grains, a few nanotwins were observed (Figure 5f) that were obviously obtained during swaging. Meanwhile, the restriction of mechanical twinning within fine recrystallized grain/sub-grains might be also derived [37] due to changes in the ratio of stresses related to the formation of perfect dislocation and Shockley partial dislocation for grains with the critical diameter [38]. The following equation might be applied to estimate the critical diameter (d_c_) for FCC alloys [37]:

(2) $$d_{c}=\frac{2\alpha \mu \left(b-b_{1}\right)b_{1}}{\gamma },$$

where μ is the shear modulus (≈80 GPa), b and b_1_ are the Burgers vectors of a complete and Shockley partial dislocations, respectively (0.248 × 10^−10^ m and √3/3 b), α is the Taylor constant (~1) and γ is the stacking fault energy (39.39 mJ/m^2^). Thus, the critical diameter was calculated at the level of ~65 nm. Hence, twinning was not restricted within the grains/sub-grains with the size of more than ~65 nm that was validated by the current work (Figure 5f).

#### 4.2.3. Stages of Bulk Gradient Structure Formation

The bulk gradient structure formation was caused by twinning in many systems with the block-like structure development in the center. Meanwhile, primary and secondary twinning, shear banding, and dynamic recovery and recrystallization occurred at the edge. Furthermore, at the late swaging degrees, dislocation cells might form cell blocks (Figure 5f) that were surrounded by dislocation boundaries to accommodate the lattice misorientation. Such differences in the mechanisms of the structure formation were associated with moderate tensile and compressive axial stresses in the center and high compressive axial stresses at the edge (Figure 9). Thus, strong two-component 111ǁBA and 001ǁBA and weak one-component 111ǁBA textures were obtained in the center and edge, respectively. Thus, the structure and texture gradients were determined by inhomogeneous plastic strain accumulation, stress distribution, and heating during swaging.

**Figure 11:**
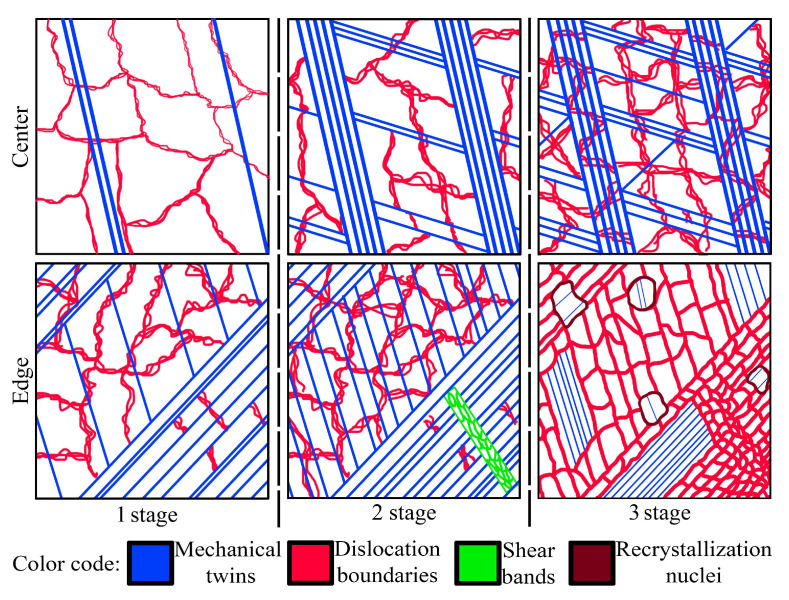
Scheme of structure evolution during swaging of the program steel. Notation: 1st stage—a 20% swaging reduction; 2nd stage—a 40–80% swaging reduction; 3rd stage—a 95% swaging reduction.

Generally, the following stages of the gradient structure formation by cold swaging might be distinguished (Figure 11): 1st stage (a 20% swaging reduction)—detached twin bundles in the center and the lamellar twin-matrix structure at the edge; 2nd stage (a 40–80% swaging reduction)—the block-like structure in the center and the lamellar twin-matrix structure partially fragmented by the formation of shear bands at the edge; 3rd stage (a 95% swaging reduction)—the block-like structure in the center and the twin-matrix structure that was fragmented by fine grains/sub-grains at the edge.

### 4.3. Mechanical Properties Analysis

#### 4.3.1. Microhardness Distribution

After swaging, the non-uniform distribution of microhardness over the rod cross-sections was observed (Figure 6). At the 1st stage of the structure evolution (after a 20% reduction), an increase in the microhardness especially at the edge was obtained. However, the microhardness distribution with the maximum in the center and the minimum at half the rod radius was observed after a reduction of 40–95% that corresponded to the 2nd and 3rd stages. Such distribution might be associated with the significant increasing residual stresses after applied swaging modes. According to Ref. [39], compressive and tensile residual stresses in the radial direction were attained in the center and edge, respectively, of the rod after swaging. Meanwhile, null residual stresses were derived at half the rod radius that caused the microhardness minimum (Figure 6).

#### 4.3.2. Tensile Properties Analysis

The stages of the structural evolution might also affect mechanical properties of the cold-swaged rod. As three stages were distinguished by analysis of the structural evolution (Section 4.2), three parts of mechanical properties plots (Figure 7b) might also be distinguished. Up to a 40% reduction, significant strengthening and loss of ductility occurred, which was associated with the enhancement of detached twin bundles in the center and the lamellar twin-matrix structure at the edge (Figure 11). The mentioned processes occurred due to accumulation and multiplication of dislocations and twins in the center and edge that resulted in strengthening and a subsequent loss of ductility.

The reduction from 40 to 80% was accompanied with the saturation of ductility (an elongation to failure of ~14%) while strengthening occurred (σ_0.2_—from ~820 to ~930 MPa; σ_B_—from ~930 to ~1020 MPa) which might be related to the effect of the gradient structure. According to Ref. [40], the effect was associated with redistribution of strain and stress between the soft center and hard edge. Therefore, plastic deformation expanded mainly in the center, while stress concentrated at the edge. Thus, strengthening and stable ductility might be obtained [7,12]. Meanwhile, austenitic stainless steels with a uniform structure obtained by dynamic plastic deformation [11] or cold rolling [8] lost ductility with an increase in strength. Despite the saturation of elongation to failure (δ), a decrease in uniform elongation (δ_U_) was also derived that was also observed in gradient materials [41]. However, after a 95% reduction, further increasing ultimate and yield strength and decreasing ductility were attained that was related to the 3rd stage of microstructure evolution.

#### 4.3.3. Strain Hardening Rate Analysis

The strain hardening rate (SHR) true strain curves (Supplementary Materials Figure S3) were calculated from the true stress-strain curves obtained by the conventional gauge-length method that was correct until the necking onset [42]. The SHR of the as-received condition was characterized by the greatest value and the longest of strain hardening ability. Meanwhile, three stages could be distinguished on the SHR plot for the as-received condition as well as after 20% reduction (Supplementary Materials Figure S3a,b). In the A stage, the strain hardening coefficient decreased with an increase in true strain. It was associated with dislocation accumulation and multiplication that might promote twin nucleus [43,44]. An increase in SHR on the B stage was associated with the twinning development because the twinning stress was a little higher than the yield strength [43]. The following C stage might be referred to the activation of the secondary twin system [44,45] when the primary twin formation was restricted [43].

On the other hand, after a reduction of 40, 60, and 80%, only A and C stages might be distinguished. However, with an increase in the swaging reduction, the interval of strain hardening narrowed and the strain hardening coefficient decreased. It should be noted that after a reduction of 20, 40, 60, and 80%, stage C demonstrated a similar level (Supplementary Materials Figure S3) that might be ascribed to the similar structure of the central part and corresponding critical stress of the activation of the secondary twin system. Importantly, after 95% of reduction, the above-mentioned three stages on the SHR plot might be found (Supplementary Materials Figure S3f). Likely, it was associated with the higher stress level that resulted in activation of twinning in uniaxial grains/sub-grains at the edge. However, it is difficult to separate swaging twins from tensile testing twins therein.

## 5. Conclusions

The effect of cold swaging on the bulk gradient structure formation and mechanical properties of the 316-type austenitic stainless steel was studied. The following results were obtained:

1. Three stages of the gradient structure formation were distinguished: 1st stage (a 20% swaging reduction)—detached twin bundles in the center and the twin-matrix structure at the edge; 2nd stage (a 40–80% swaging reduction)—the block-like structure in the center and the twin-matrix structure fragmented by shear bands at the edge; 3rd stage (a 95% swaging reduction)—the block-like structure in the center and the twin-matrix structure fragmented by fine sub-grains formation at the edge.
2. The strong two-component 111ǁBA and 001ǁBA and weak one-component 111ǁBA textures in the center and edge, respectively, were developed during swaging. In the center, the fraction of 111ǁRA-oriented grains reached a maximum of 63% after a 60% reduction with a following decrease to 51% after an 80% reduction, while the fraction of 001ǁRA-oriented grains increased steadily. The fraction of 111ǁRA- and 001ǁRA-oriented grains at the edge did not exceed 24–27%.
3. Swaging resulted in non-uniform strain accumulation. Higher plastic strain was accumulated in the surface layers compared to the rod center. Meanwhile, moderate tensile and compressive axial stresses were obtained in the center during cold swaging. Yet, high compressive axial stresses were derived at the edge. The increase of cold swaging reduction led to the development of dislocation cells and cell blocks that promote additional strengthening factors for the gradient structured materials.
4. Until a 40% reduction, significant tensile strengthening and loss of ductility occurred. After the 40, 60, and 80% reduction, the saturation of ductility (δ–14%) with strengthening occurred (σ_0.2_—from ~820 to ~930 MPa; σ_B_—from ~930 to ~1020 MPa). After a 95% reduction, further increasing ultimate and yield strength and decreasing ductility were obtained.

## Data Availability

Not applicable.

## Supplementary Materials

The following are available online at https://www.mdpi.com/article/10.3390/ma15072468/s1, Figure S1: Longitudinal TEM structure after different swaging modes. Figure S2: Heating of the rod during swaging at (a) the 1st (20%), (b) 2nd (40%), (c) 3rd (60%), (d) 4th (80%), and (e) 5th (95%) steps of the processing. Figure S3: True stress-true strain and dσ/dε-true strain plots for the program steel in (a) the as-received condition and after (b) a 20%, (c) 40%, (d) 60%, (e) 80%, and (f) 95% swaging reduction (SR).
