# Supplementary material for: Effect of Cold Swaging on the Bulk Gradient Structure Formation and Mechanical Properties of a 316-Type Austenitic Stainless Steel
Source: Materials (Basel). 2022 Mar 27;15(7):2468. doi: 10.3390/ma15072468 (PMC8999866; doi:10.3390/ma15072468)
Supplement: Supplementary file 1 [file materials-15-02468-s001.zip › materials-1628924-supplementary.pdf]

Supplementary materials

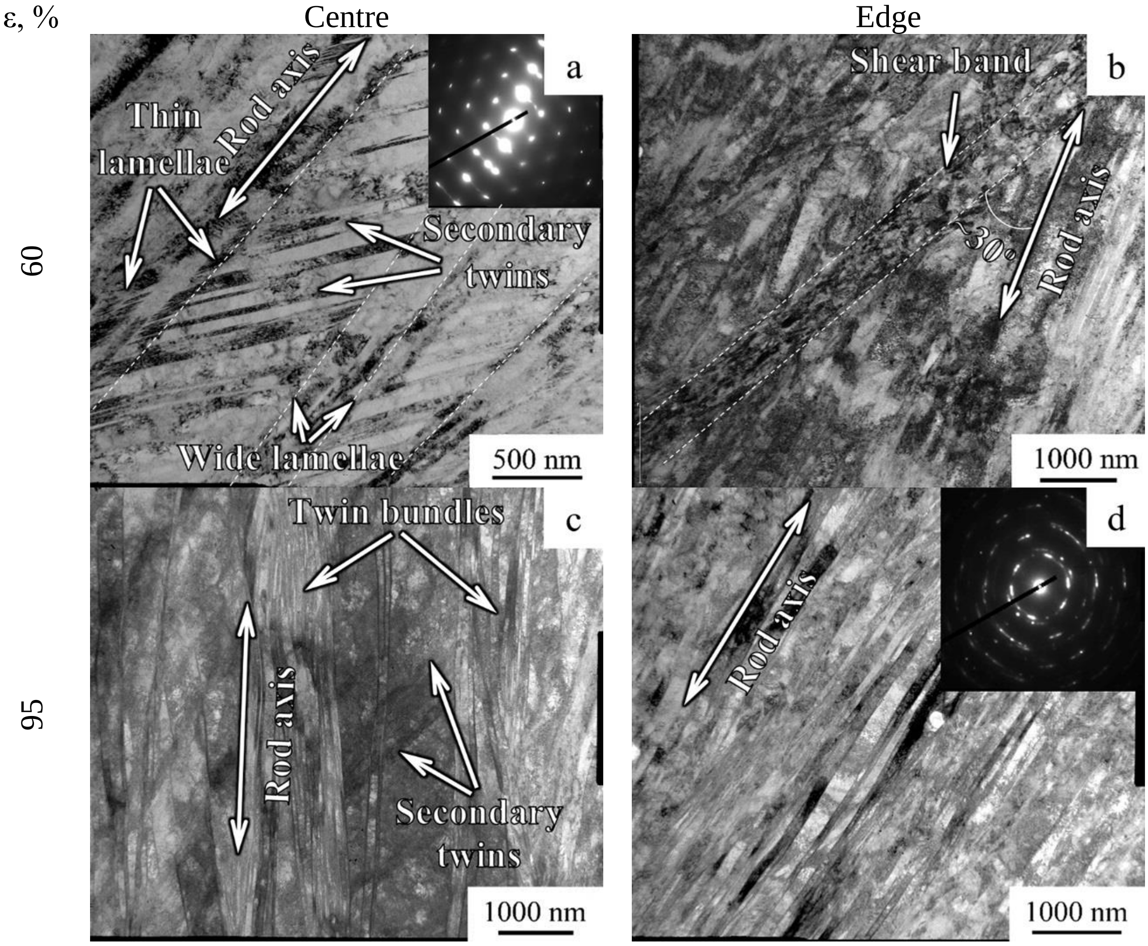

Figure S1. Longitudinal TEM structure after different swaging modes.

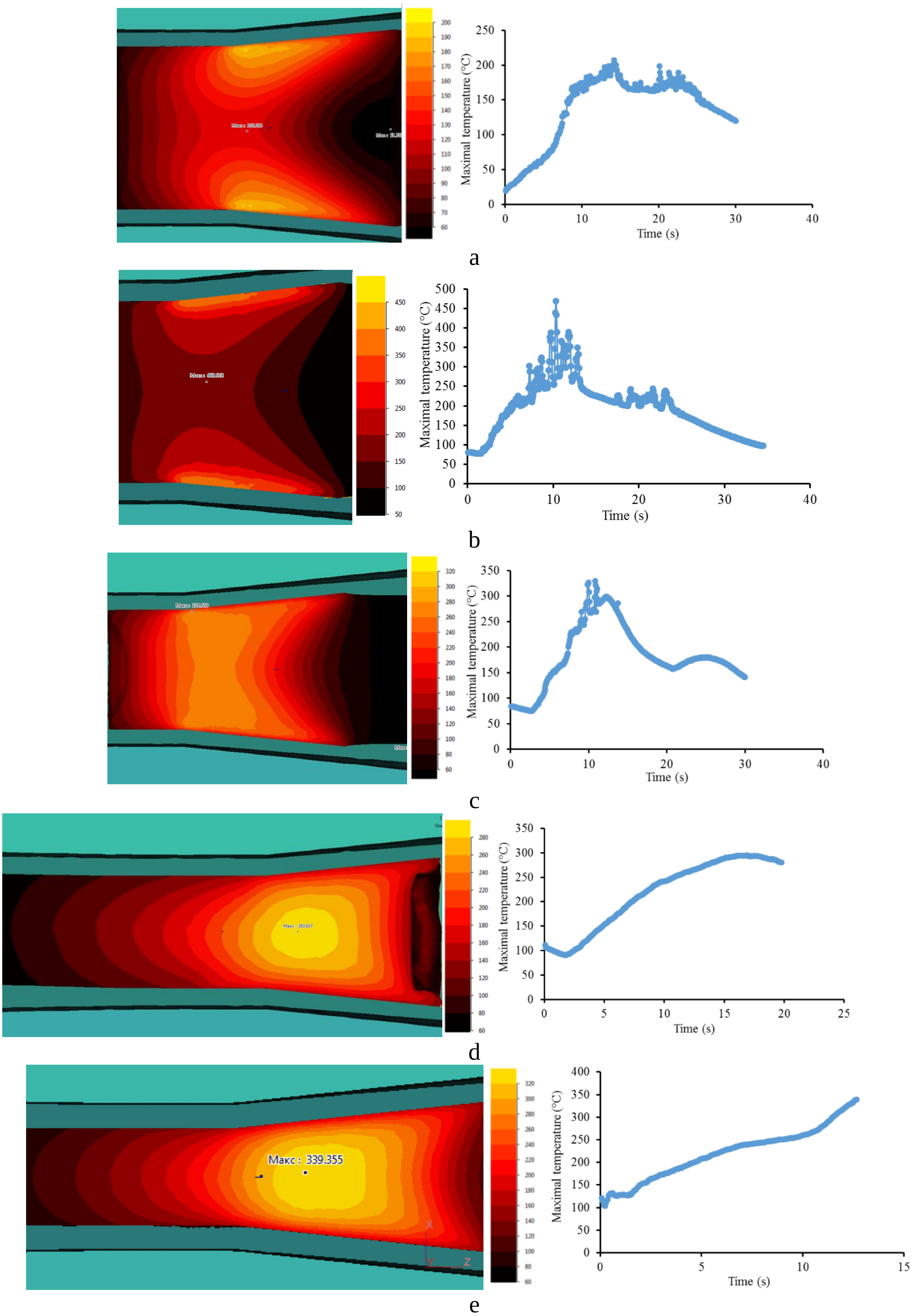

Figure S2. Heating of the rod during swaging at (a) the 1st (20%), (b) 2nd (40%), (c) 3rd (60%), (d) 4th (80%), and (e) 5th (95%) steps of the processing.

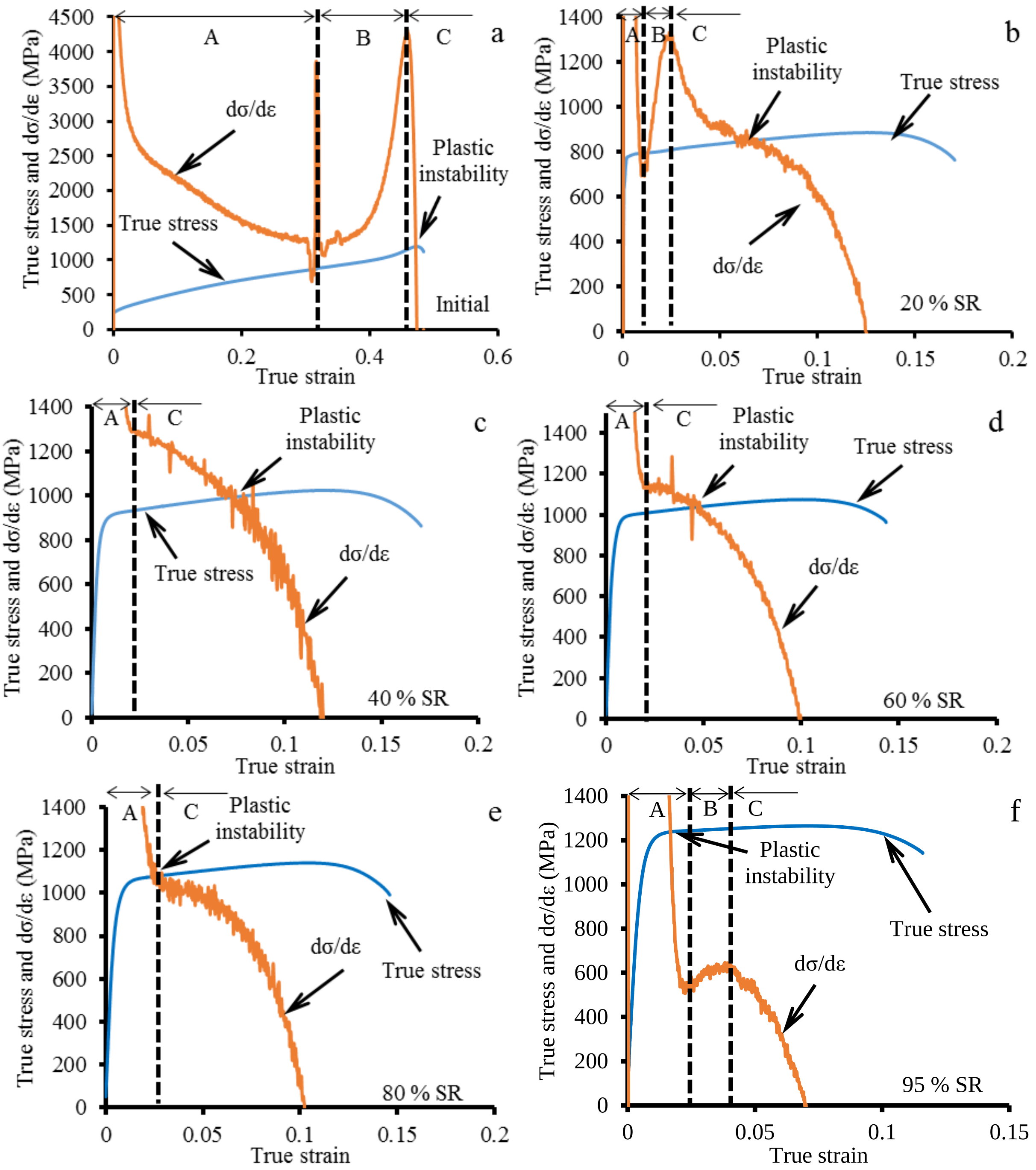

Figure S3. True stress-true strain and dσ/dε-true strain plots for the program steel in (a) the as-received condition and after (b) a 20%, (c) 40%, (d) 60%, (e) 80%, and (f) 95% swaging reduction (SR).
